# Cellular evidence for nano-scale exosome secretion and interactions with spermatozoa in the epididymis of the Chinese soft-shelled turtle, *Pelodiscus sinensis*

**DOI:** 10.18632/oncotarget.8092

**Published:** 2016-03-15

**Authors:** Hong Chen, Ping Yang, Xiaoya Chu, Yufei Huang, Tengfei Liu, Qian Zhang, Quanfu Li, Lisi Hu, Yasir Waqas, Nisar Ahmed, Qiusheng Chen

**Affiliations:** ^1^ Laboratory of Animal Cell Biology and Embryology, College of Veterinary Medicine, Nanjing Agricultural University, Nanjing, Jiangsu Province, China

**Keywords:** Chinese soft-shelled turtle, nano-scale exosomes, spermatozoa, epididymis, interaction, Pathology Section

## Abstract

The epididymis is the location of sperm maturation and sperm storage. Recent studies have shown that nano-scale exosomes play a vital role during these complicated processes. Our aim was to analyze the secretory properties of epididymal exosomes and their ultrastructural interaction with maturing spermatozoa in the Chinese soft-shelled turtle. The exosome marker CD63 was primarily localized to the apices of principal cells throughout the epididymal epithelium. Identification of nano-scale exosomes and their secretory processes were further investigated via transmission electron microscopy. The epithelium secreted epididymal exosomes (50~300 nm in diameter) through apocrine secretion and the multivesicular body (MVB) pathway. Spermatozoa absorbed epididymal exosomes through endocytosis or membrane fusion pathways. This study shows, for the first time, that nano-scale exosomes use two secretion and two absorption pathways in the reptile, which may be contribute to long-term sperm storage.

## INTRODUCTION

After spermiation in the testis, spermatozoa are functionally incompetent and are unable to fertilize an oocyte even though they are morphologically mature. Fertilization ability is acquired during spermatozoa transit through the epididymis [1, 2]. The epididymis is a single, long, convoluted tubule that is usually divided into three main segments based on their anatomical properties: caput, corpus, and cauda [3]. Epididymal transit lasts 5-10 days in the mouse [4] and leads to the acquisition of sperm motility and the ability to recognize and fertilize oocytes [5]. Unlike mammals, most reptile species, such as snakes [6] and turtles [7], have more obvious sperm storage [8] in the epididymis. In our previous work, we found that the Chinese soft-shelled turtle *Pelodiscus sinensis* has typical long-term sperm storage in the epididymis [9, 10]. Spermatogenesis ends with spermiation, when spermatozoa are released into the epididymis in a single event in late October [11]. Immature spermatozoa are transferred into the epididymis and stored until the following reproductive season (from May to October) [12]. This reproductive strategy is necessary for *P. sinensis* to successfully breed.

When spermatozoa migrate to the epididymis, chromatin in the sperm head condenses and the process of transcription and translation arrests. Sperm maturation and modification require the interaction between male gamete and their microenvironment in the epididymis [13]. The epididymal fluid milieu is comprised of organic and inorganic compounds generated by epithelial secretion and reabsorption [7, 14]. While in the epididymis, spermatozoa are exposed to different secretions that modulate their metabolism, their membrane and intracellular structures, and their biochemical composition [15]. The most important maturational changes of spermatozoa involve plasma membrane remodeling and the acquisition of forward motility [2]. These changes are primarily the result of proteins and lipids acquired by the spermatozoa plasma membrane [16, 17]. Intercellular communications are performed by membrane vesicle secretion and uptake in many biological systems. These vesicles can be classified as exosomes, ectosomes, microvesicles, apoptotic vesicles, and other extracellular vesicles according to their size, functions and cellular origin [18]. Exosomes are membrane-bound nano-particles secreted from cells under both physiological and pathological conditions, and they are released into the extracellular environment upon multivesicular body (MVB) fusion with the plasma membrane [19, 20]. Exosomes have always been defined as 30-100 nm in diameter, but a recent study has shown that exosomes from different histological ovarian epithelial cancer cells differ in size [21]. Exosomes are conserved structures found in every cell type [22] and were identified many years ago [23]. With the exception of protein and lipid transfer, exosomes are a novel mechanism for the genetic exchange between cells by delivering mRNAs and microRNAs [24]. Extracellular vesicles and their miRNA cargo are transferred to recipient cells after endocytosis or membrane fusion [25]. Due to their ability to transfer nucleic acids, the study of extracellular vesicles in oncology is a rapidly evolving and expanding field [26-28].

Similar to other biological fluids, epididymal fluids include nano-scale exosomes that measure 50~300 nm in diameter. These vesicles are also called epididymosomes and are released from epididymal principal cells [29]. Epididymal exosomes play a major role in sperm maturation by transferring proteins important for acquiring fertilization ability and forward motility [15, 30]. The presence of small membrane vesicles, or nano-scale exosomes, has been described in rats [31], mice [32], and bovine [33],but there has not yet been a report of the presence of membranous vesicles in the reptile epididymis. The majority of studies have focused on exosomes isolated from cell culture fluid and adopted *in vitro* models to study the mechanisms of secretion and absorption [33, 34].

In this study, we used the Chinese soft-shelled turtle to investigate nano-scale exosomes in sperm maturation and sperm storage. To our knowledge, this is the first *in vivo* experiment to substantiate the secretion pathway of epididymal exosomes in the reptile. We also examined the ultrastructural characteristics between nano-scale exosomes and maturing spermatozoa.

## RESULTS

### CD63 expression in the epididymis

**Figure 1:**
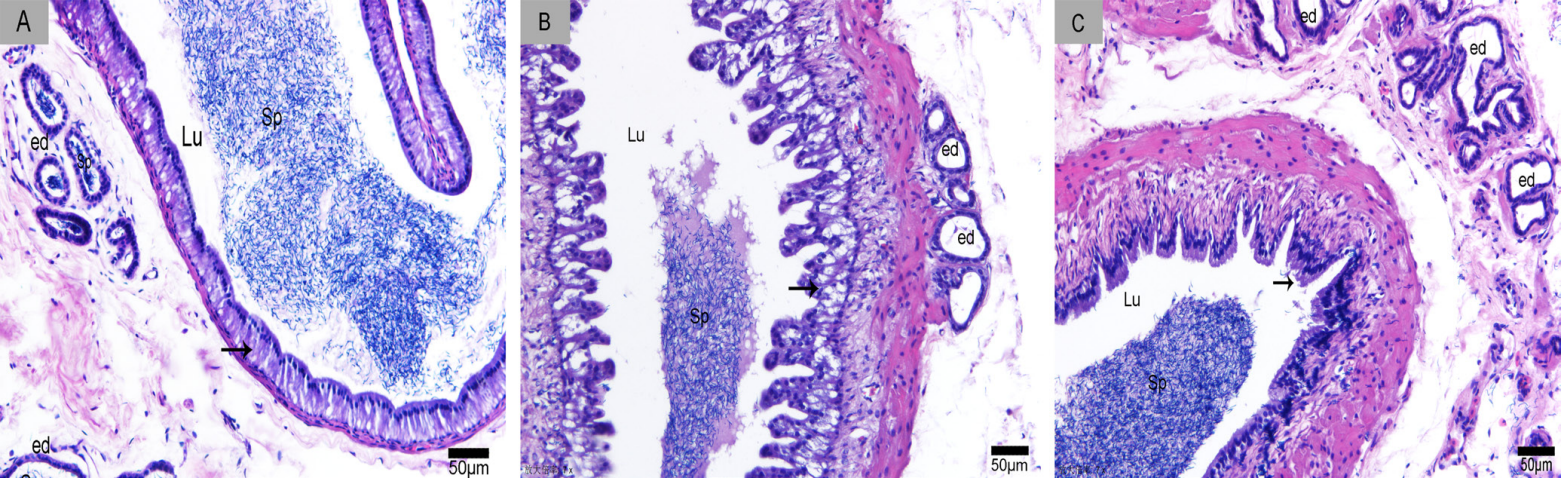
Sperm storage in the epididymis **A.** caput, **B.** corpus and **C.** cauda. Lu: lumen; Sp: spermatozoa; arrowhead: epididymal epithelium; ed: efferent duct; H & E stain; Bars: A, B, C = 50 μm.

**Figure 2:**
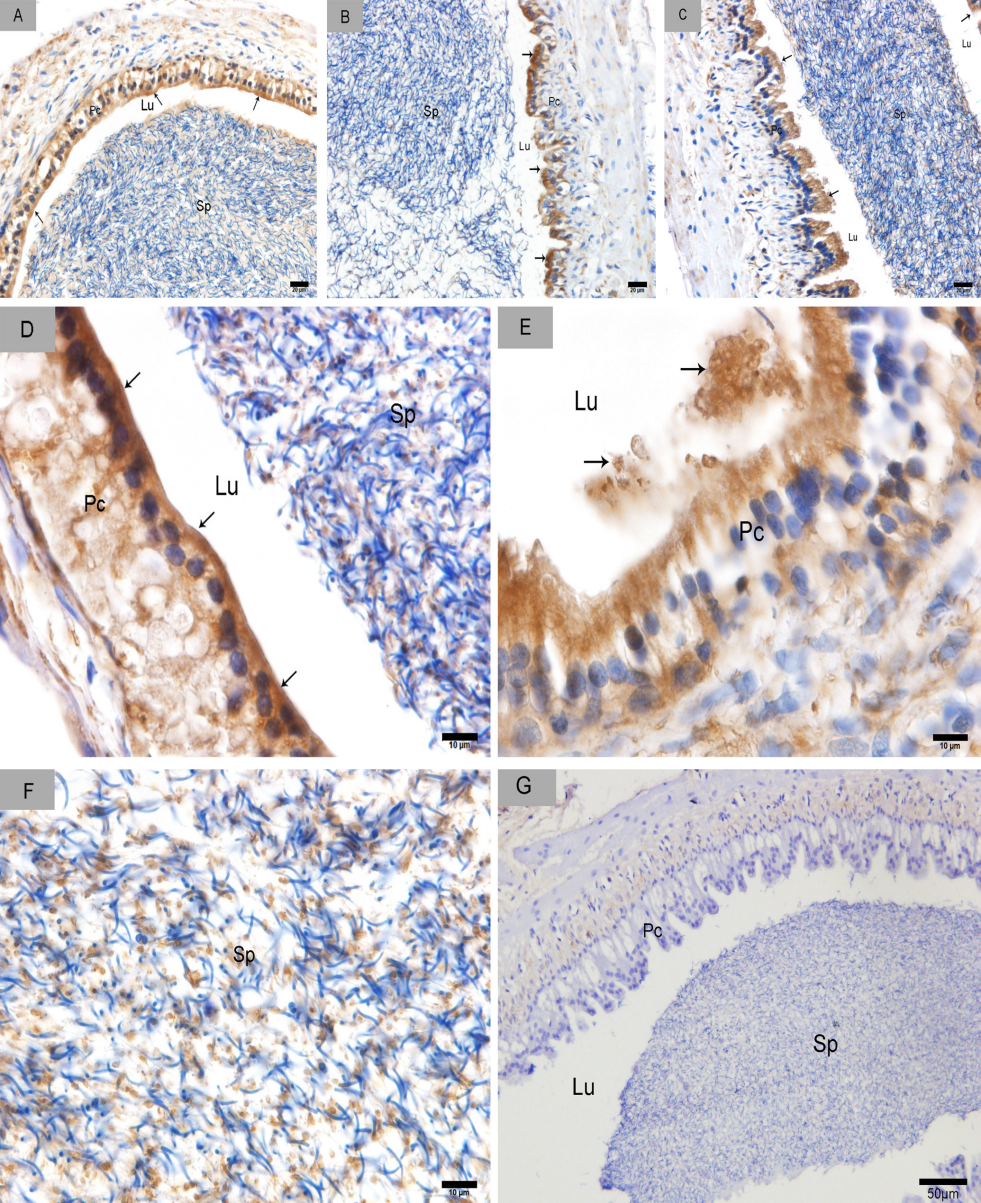
CD63 immunohistochemistry in the epididymis Arrowhead shows positive CD63 expression. **A.** caput (Nov), **B.** corpus (Apr), **C.** cauda (July), **D.** caput (Nov), **E.** corpus (Apr), **F.** cauda (July) and **G.** Negative control. Sp: spermatozoa; Lu: lumen; Pc: Principal cell; Bars: A, B, C = 20 μm; D, E, F = 10 μm; G = 50 μm.

We divided the Chinese soft-shelled turtle epididymis into three regions: caput, corpus and cauda. The epididymis was lined with pseudostratified epithelium primarily composed of principal and basal cells (Figure 1). CD63-positive cells were distributed in the caput (Figure 2A), corpus (Figure 2B) and cauda (Figure 2C). The epididymal lumen was filled with spermatozoa at different stages of sperm storage. We observed the strongest immunoreactivity at the apices of principal cells (Figure 2D). Some CD63-positive structures appeared to be shed from principal cells (Figure 2E). There were many positive particles scattered between spermatozoa (Figure 2F). CD63 expression was similar at early Nov, April and July, respectively, which corresponded to the early, intermediate and late stages of sperm storage in the epididymis.

### TEM detection of nano-scale epididymal exosomes

**Figure 3:**
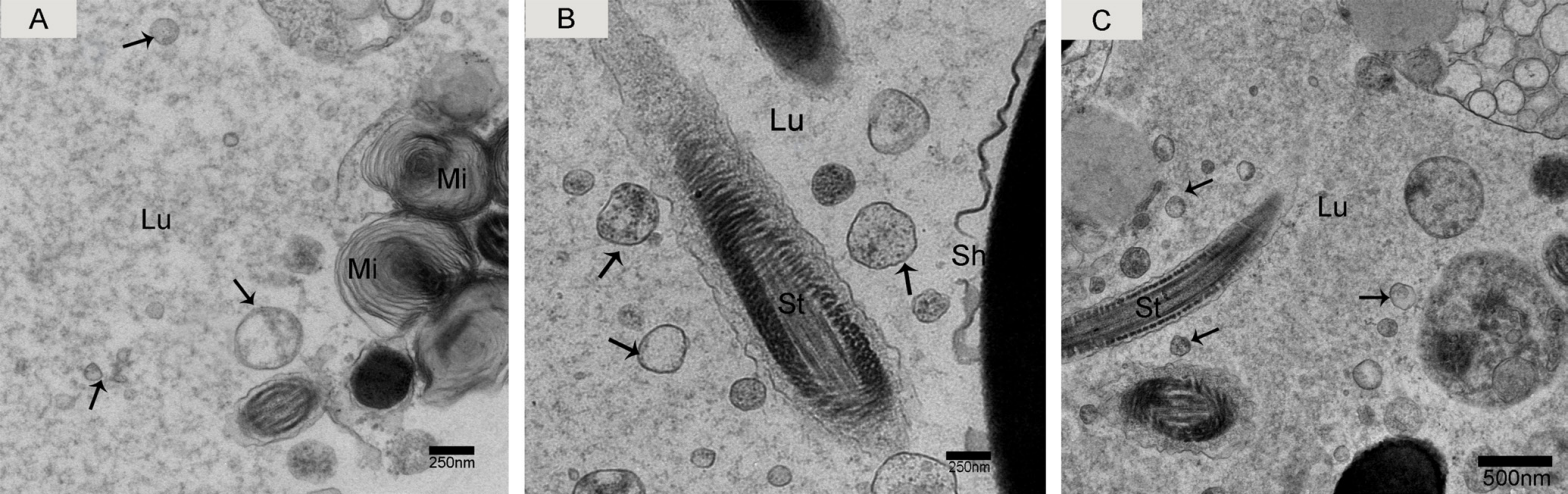
Electron micrograph of nano-scale exosomes (arrowhead) around spermatozoa in the epididymal lumen **A.** caput, **B.** corpus, **C.** cauda. Lu: lumen; Mi: mitochondria; St: sperm tail; Sh: sperm head; Bars: A, B = 250 nm; C = 500 nm.

**Figure 4:**
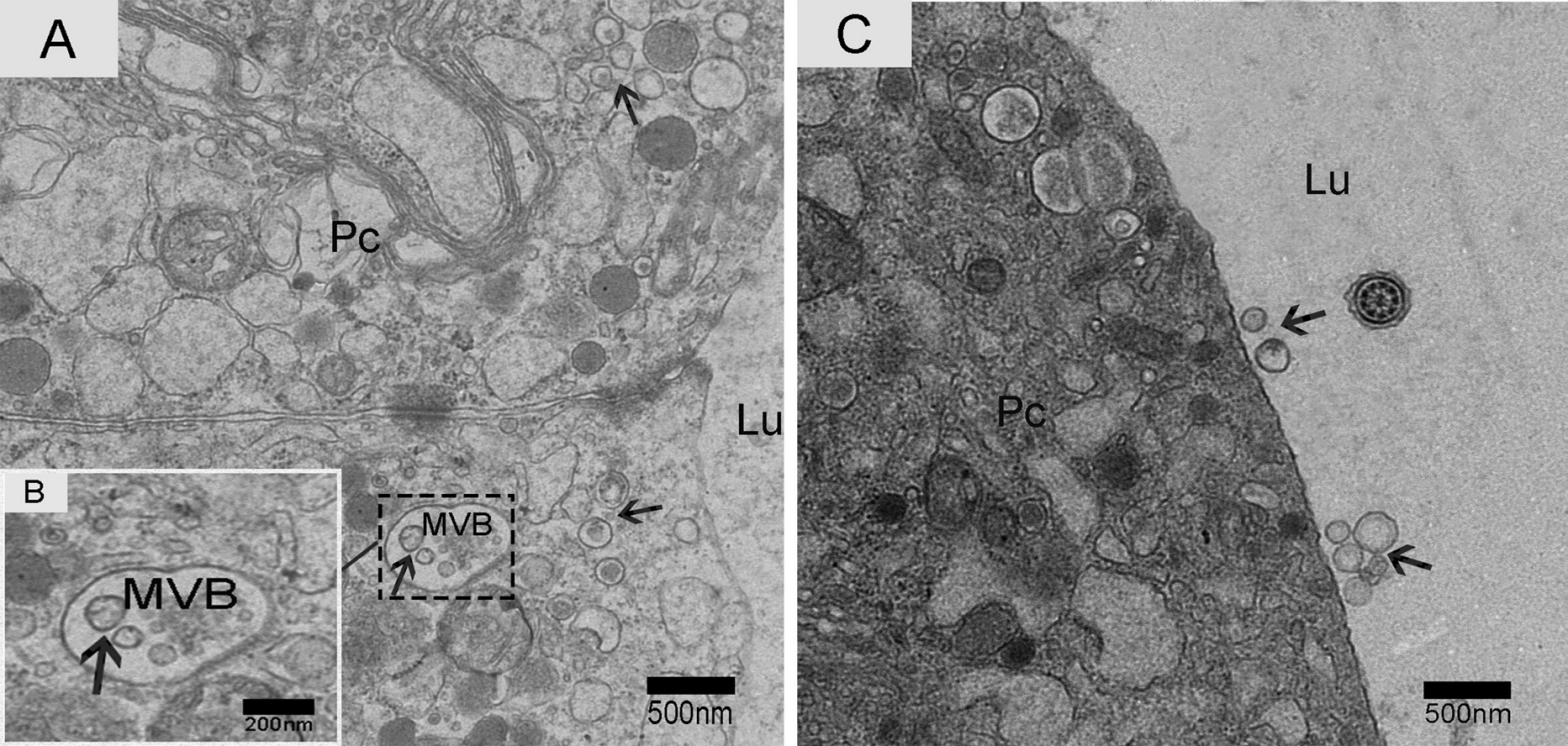
Electron micrograph of nano-scale exosomes Nano-scale exosomes (arrowhead) are scattered in the principal cells or enclosed in MVB. **A.** Zoomed-in image of MVB containing nano-scale exosomes. **B.** Nano-scale exosomes (arrowhead) are close to the principal cell surface. **C.** Pc: principal cell; MVB: multivesicular body; Lu: lumen; Bars: A, C= 500 nm; B = 200 nm.

**Figure 5:**
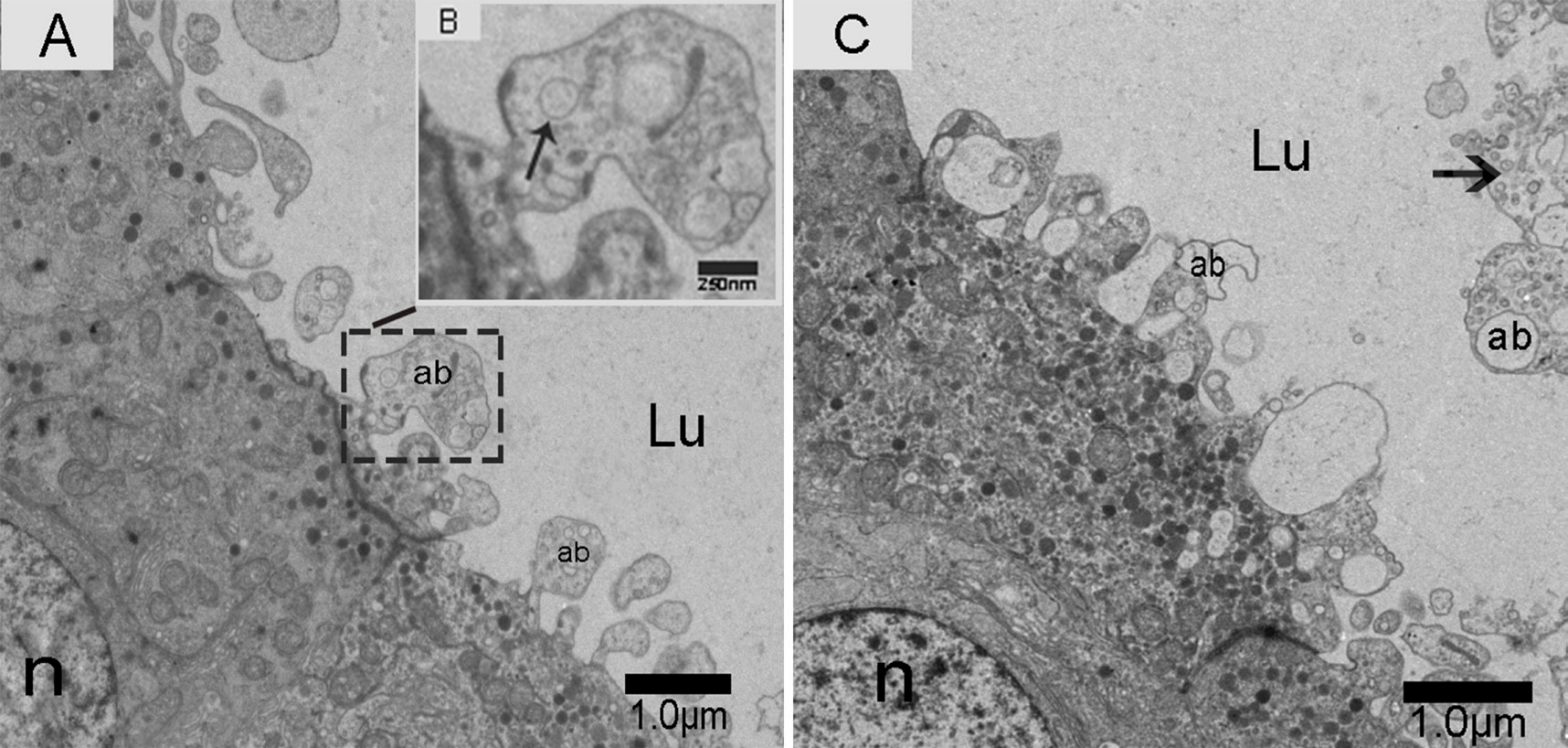
Electron micrograph of nano-scale exosomes (arrowhead) released by the epithelium Apical blebs formed on the principal cell surface. **A.** Zoomed-in image of apical blebs containing nano-scale exosomes (arrowhead). **B.** Nano-scale exosomes (arrowhead) are released into the lumen. **C.** Lu: lumen; n: nucleus; ab: apical blebs; Bars: A, C = 1.0 μm; B = 250 nm.

We observed nano-scale exosomes in the lumen of all three epididymal regions (Figure 3), which ranged from 50-300 nm in diameter. We also detected them within (Figure 4A) and outside of (Figure 4C) the epithelium. Interestingly, multivesicular body (MVB) associated with nano-scale exosomes (Figure 4B) was located in the epididymal epithelial cells (Figure 4A). In addition, some apical blebs containing many nano-scale exosomes localized to the epithelial surface (Figure 5A). These blebs detached from the epithelial surface. Finally, as a consequence of fragmentation, the contents of the blebs were released into the epididymal lumen (Figure 5C).

### Interactions between nano-scale exosomes and spermatozoa in the epididymis

**Figure 6:**
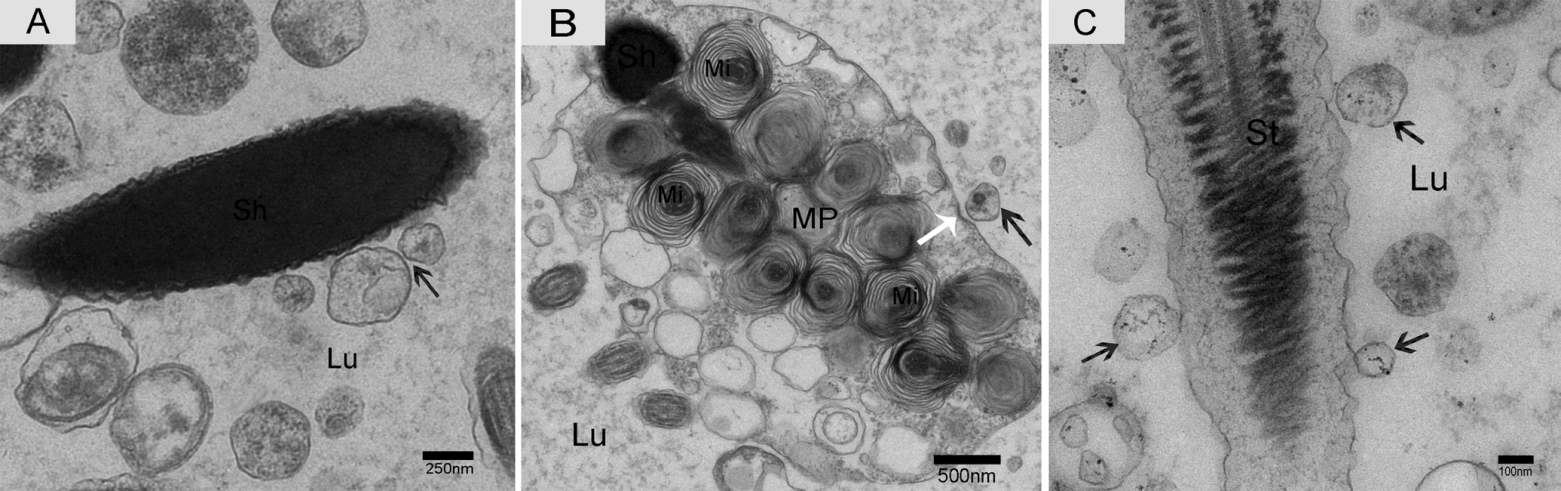
Electron micrograph of nano-scale exosomes and spermatozoa in the epididymis Nano-scale exosomes (arrowhead) contacted the spermatozoa head **A.**, midpiece **B.** and principal piece **C.** The corresponding cytoplasmic membrane (white arrow) is depressed (B). Lu: lumen; Sh: sperm head; MP: midpiece; St: sperm tail; Mi: mitochondria; Bars: A = 250 nm, B = 500 nm, C = 100 nm.

**Figure 7:**
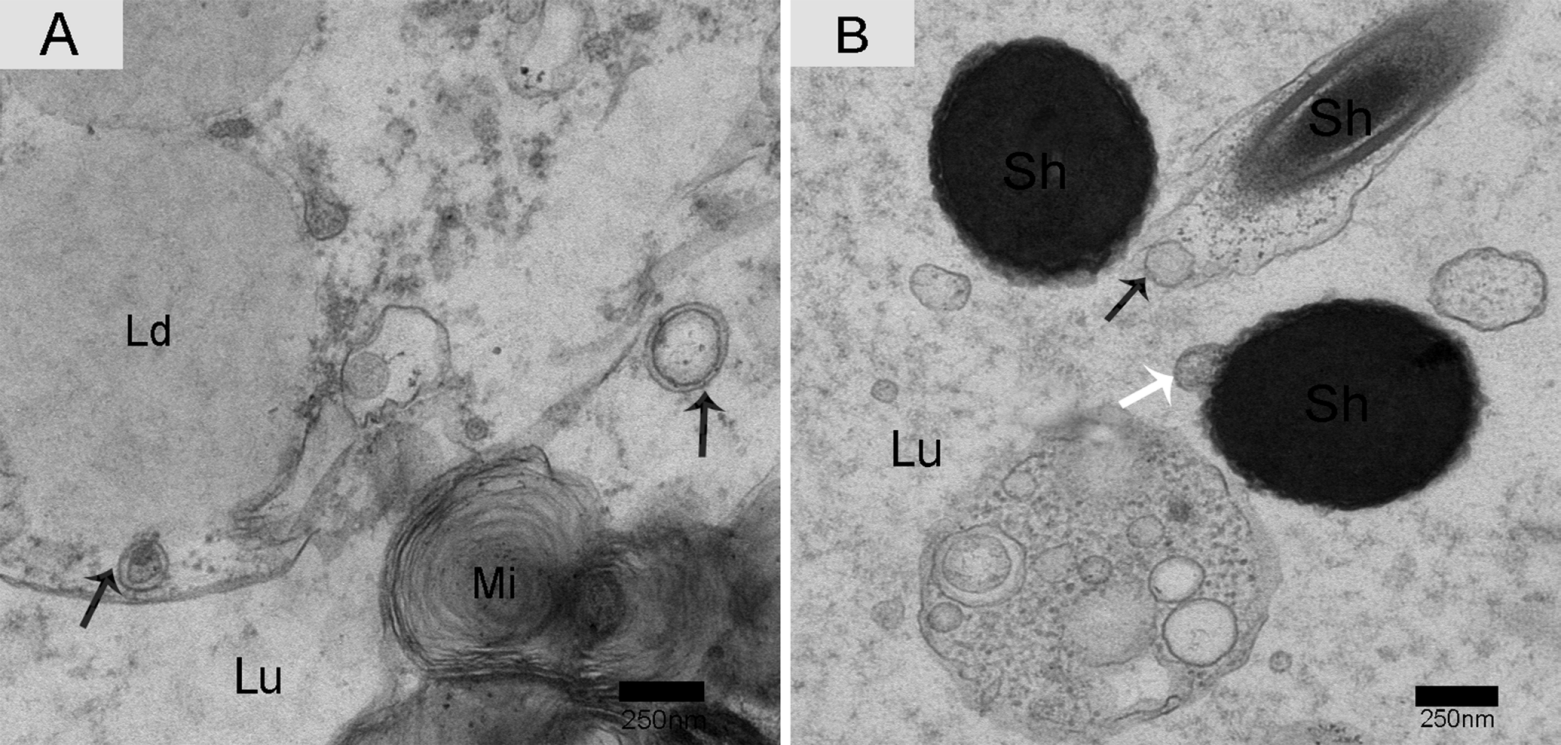
Electron micrograph of nano-scale exosomes with double-layer membranes and absorption in the epididymis Nano-scale exosomes (arrowhead) have a double-layered membrane. **A.** Nano-scale exosomes were taken up by the endocytic pathway (arrowhead) or through membrane fusion (white arrow). **B.** Lu: lumen; Sh: sperm head; Mi: mitochondria; Ld: lipid droplets; Bars: A, B = 250 nm.

Nano-scale exosomes were distributed among spermatozoa throughout the lumen of the epididymis, and some made contact with the sperm head (Figure 6A). When the nano-scale exosomes were next to the spermatozoa midpiece, the corresponding cytoplasm membrane was depressed (Figure 6B). We also observed that the principal piece membrane in maturing spermatozoa morphologically contacted with the nano-scale exosomes within the lumen (Figure 6C). Some nano-scale exosomes localized to spermatozoa cytoplasm and the lumen, and these nano-scale exosomes showed a clear double-layer membrane structure (Figure 7A). The nano-scale exosome membrane fused with the spermatozoa membrane, and sometimes the entire nano-scale exosome was internalized by the spermatozoa *via* endocytosis (Figure 7B).

## DISCUSSION

Cell-cell communication is a very complicated mechanism for information exchange. Direct membrane contact is the most evident way for two cells to communicate [35]. However, some soluble secreted molecules have also been shown to be important mediators of information transmission, including cytokines, hormones, and bioactive lipids. An increasing number of studies have shown a third mechanism for cell-cell communication where exosomes (30~100 nm in diameter) released by one cell can transmit essential information to its target cells [36-38]. Thus, all nano-scale exosomes secreted from cells under both physiological and pathological conditions should be recognized as a new modulatory system, just like the neural and humoral regulation systems. Here, we used the Chinese soft-shelled turtle epididymis as a model to explore the roles of nano-scale exosomes during sperm storage and maturation. Our results revealed that nano-scale exosomes can constitute the microenvironment of the epididymis to allow for long-term sperm storage, which is important for each step of sperm maturation in the epididymis.

The epididymis has an extraordinary complexity at both the structural and functional levels [39]. The segmented gene expression patterns throughout the epididymal duct of mammals results in distinct secretions in the intraluminal milieu and the formation of microenvironments optimal for each step of sperm maturation [40]. Thus, the three epididymal segments can secrete different exosomes into the lumen of mammals. Our previous study showed that the efferent ducts are present throughout all epididymal regions, which is a typical feature of the Chinese soft-shelled turtle [9]. If the epididymis is important in sperm storage and maturation, then the spermatozoa would be in the efferent ducts down the entire length of the epididymis. The three epididymal segments have similar abilities in sperm storage, which is distinct from mammals. In this study, we found that CD63 expression was similar in each of the three epididymal regions. This is consistent with the ability of each epididymal segment to store sperm. The epithelium releases different size nano-scale exosomes to facilitate spermatozoa maturation at different stages of sperm storage. This is likely why we saw similar CD63 expression at the three time points.

Only one secretion pathway involved in nano-scale exosomes has been reported in the mammalian epididymis. Previous analysis revealed that the epididymal epithelium was capable of apocrine secretion [41]. Apocrine secretion is characterized by the formation of apical blebs by the epithelium, where the apical blebs detach from the apical membrane and release their contents into the lumen through fragmentation [42-44]. We performed TEM analysis, which is considered the gold standard for exosome identification [45, 46]. We found that the epididymal exosomes in the Chinese soft-shelled turtle were round in shape and approximately 50-300 nm in size, which was similar to those previously described in mammals [33, 42, 47]. Importantly, TEM results provided significant evidence for apocrine secretion of nano-scale exosomes. We also detected multivesicular body (MVB) in the principal cells of Chinese soft-shelled turtles. MVB fusion with the plasma membrane led to the release of the internal vesicles into the extracellular space. There was a clear significant difference between the above two nano-scale exosome secretion pathways. Based on these observations, we hypothesized that the principal cells secrete nano-scale exosomes into the epididymal lumen by apocrine secretion and MVB pathways.

There are currently two known mechanisms of exosome generation in tumour cells. The first model is that exosomes formed *via* the endocytic pathway in a two-step process and are released into the extracellular environment from the plasma membrane *via* multivesicular body (MVB) [48, 49]. The second means of secretion is the ESCRT-independent mechanism [50], and this method was shown to require the sphingolipid ceramide (Cer) [51]. Therefore, our results provide a new research direction for tumour-derived exosomes.

Information transfer can occur through several mechanisms, while direct contact is a very important basic method [24]. The ultrastructure in the Chinese soft-shelled turtle indicates that nano-scale exosomes contact the head, midpiece and principal piece of spermatozoa within the epididymal lumen. TEM results gave clear evidence for two different absorption pathways of nano-scale exosomes. One pathway involves nano-scale exosome membrane fusion within the spermatozoa membrane, and the other pathway is through the endocytic pathway (as shown in Figure 7B). Analysis of their interaction and absorption could provide direct evidence for the roles of epididymal exosomes, which may act as messengers for epithelium-spermatozoa communication and signal transduction. Meanwhile, nano-scale exosome absorption and contact with different regions of spermatozoa are very important factors for long-term sperm storage in the epididymis of Chinese soft-shelled turtles.

In summary, the apical surface of epididymal epithelial cells secreted nano-scale exosomes into the luminal fluids. Furthermore, principal cells released epididymal exosomes through apocrine secretion and MVB pathways. We provided significant evidence for the contact and absorption of nano-scale exosomes by spermatozoa. Chinese soft-shelled turtle may be an *in vivo* model for investigating and obtaining nano-scale exosomes.

## MATERIALS AND METHODS

### Animals

The Chinese soft-shelled turtle *Pelodiscus sinensis* is one of the most representative classes of reptiles that hibernate (from Dec to Apr). Sexually mature male Chinese soft-shelled turtles were purchased in early November, April and July from a pond in Nanjing, south China. Five turtles were taken at random from the pond each time, anaesthetized by intraperitoneal administration of sodium pentobarbital (20 mg/kg) and sacrificed by cervical dislocation. Five epididymis pairs were used each time, and all efforts were made to minimize animal suffering. The protocol was approved by the Science and Technology Agency of Jiangsu Province (SYXK (SU) 2010-0005).

### Light microscopy

Samples were fixed in 10% neutral buffered formalin (v/v) overnight, embedded in paraffin wax, and serial sectioned (5 μm). A section of each region of the epididymis (caput, corpus and cauda) was stained with haematoxylin and eosin (H & E) for light microscopic observation.

### Immunohistochemistry

Three different month epididymis of the Chinese soft-shelled turtle were examined by immunohistochemistry, which showed surface protein CD63 expression on exosomes, as it is a commonly used exosome marker [52]. Three tissue slides containing 5 μm sections of the epididymis (caput, corpus and cauda) were processed through a standard immunohistochemistry protocol as described previously [53]. The rabbit anti-CD63 antibody (ab134045, Abcam, Cambridge, UK) was used at a dilution of 1:75. Sections incubated in PBS alone served as negative controls.

### Transmission electron microscopy (TEM)

Three epididymal sample regions were cut into small blocks and fixed in 2.5% (v/v) glutaraldehyde in phosphate-buffered saline (PBS; 4°C, pH 7.4, 0.1 M) for 24 h. The samples were rinsed in PBS, post-fixed in 1% (w/v) osmium tetroxide in the same buffer for 1 h and washed in buffer. The samples were dehydrated in increasing concentrations of ethyl alcohol and infiltrated with a propylene oxide-Araldite mixture for embedding in Araldite. The blocks were sectioned, and ultrathin sections (50 nm) were mounted on Formvar-coated grids, and stained with uranyl acetate and lead citrate for 20 min per step. The sections were examined and photographed using a transmission electron microscope (TEM; Hitachi H-7650, Japan).
